# Effects of Different Doses of Ranolazine on SIRT1, APELA, and APL13 in a Rat MCAO Model

**DOI:** 10.3390/cimb48060609

**Published:** 2026-06-10

**Authors:** Abdulkadir Kankilic, Ibrahim Basar, Selim Karahan, Ulas Alabalik, Revsa Evin Canpolat Erkan, Omer Karakoyun, Ismail Yildiz, Mehmet Yigit Akgun, Ozkan Ates, Meral Erdinc

**Affiliations:** 1 Department of Neurosurgery, Faculty of Medicine, Dicle University, 21280 Diyarbakır, Türkiye; dr.abdulkadirkankilic@gmail.com (A.K.);; 2 Department of Medical Pharmacology, Faculty of Medicine, Mardin Artuklu University, 47200 Mardin, Türkiye; 3 Department of Pathology, Faculty of Medicine, Dicle University, 21280 Diyarbakır, Türkiye; 4 Department of Biochemistry, Faculty of Medicine, Dicle University, 21280 Diyarbakır, Türkiye; 5 Department of Dermatology and Venereology, Gazi Yaşargil Training and Research Hospital, 21010 Diyarbakır, Türkiye; 6 Department of Biostatistics, Faculty of Medicine, Dicle University, 21280 Diyarbakır, Türkiye; 7 Department of Neurosurgery, Faculty of Medicine, Koç University, 34010 Istanbul, Türkiye; 8 Department of Medical Pharmacology, Faculty of Medicine, Dicle University, 21280 Diyarbakır, Türkiye

**Keywords:** ranolazine, MCAO, neuroprotection, APELA, APL13, SIRT1

## Abstract

This study investigated the effects of different doses of ranolazine in a middle cerebral artery occlusion/reperfusion (MCAO-I/R) model by evaluating histopathological changes and serum Sirtuin 1 (SIRT1), Apela peptide (APELA), and Apelin-13 (APL13) levels. A total of 47 male Sprague Dawley rats (250 ± 20 g) were randomly assigned to five groups: Sham (*n* = 7), MCAO (*n* = 10), MCAO+RAN10 (*n* = 10), MCAO+RAN30 (*n* = 10), and MCAO+RAN50 (*n* = 10). MCAO-I/R was induced by transient filament occlusion of the right middle cerebral artery for 90 min followed by reperfusion. Ranolazine was administered intraperitoneally once daily for 21 days in the treatment groups. Serum SIRT1, APELA, and APL13 levels were measured using enzyme-linked immunosorbent assay (ELISA), and brain tissues were evaluated histopathologically for neuronal degeneration and apoptotic cell counts. Histopathological analysis revealed significant neuronal degeneration and increased apoptosis in the MCAO group compared with the Sham group. Ranolazine treatment did not demonstrate significant histopathological improvement compared with the untreated MCAO group. Among the treatment groups, the MCAO+RAN50 group showed higher apoptotic cell counts and lower serum biomarker levels than the other ranolazine-treated groups. Serum SIRT1, APELA, and APL13 levels were lowest in the MCAO+RAN50 group, with selected pairwise differences reaching statistical significance. Under the present experimental conditions, clear evidence of neuroprotection could not be demonstrated. None of the ranolazine-treated groups showed significant histopathological improvement compared with the untreated MCAO group. These findings indicate that higher-dose ranolazine was not associated with neuroprotection under the conditions of this study. However, given the limited sample size, absence of infarct volume analysis, lack of neurological functional assessment, and absence of tissue-level molecular validation, further studies are required to clarify the biological significance and potential clinical relevance of the observed biomarker changes.

## 1. Introduction

Stroke remains one of the leading causes of death and disability worldwide. According to recent global estimates, there were 93.8 million prevalent stroke cases and 11.9 million incident stroke cases in 2021, with ischemic stroke accounting for approximately 65% of all incident stroke events. According to the World Health Organization, stroke was the third leading cause of death and disability worldwide in 2021 [1]. Ischemic stroke most commonly results from thrombotic or embolic occlusion of cerebral arteries, and the middle cerebral artery is one of the most frequently affected vessels [2].

Neurons are highly sensitive to hypoxia because of their substantial energy demands. Cerebral ischemia disrupts Adenosine Triphosphate (ATP) synthesis through oxygen and glucose deprivation, leading to failure of energy-dependent cellular functions and initiation of the ischemic cascade, which may ultimately result in cell death. The severity, duration, and localization of ischemia determine the extent of neuronal injury [3]. In contrast to the irreversibly damaged ischemic core, neurons within the surrounding penumbra region remain electrically silent but potentially viable. If timely reperfusion is achieved, this tissue may be salvaged, making preservation of the penumbra a major therapeutic goal in acute ischemic stroke [4]. Increased apoptosis has been recognized as one of the principal mechanisms contributing to ischemia-related neuronal loss [5,6,7].

Current treatment strategies for acute ischemic stroke focus on rapid reperfusion, neuroprotection, prevention of secondary injury, and management of complications. Recanalization can be achieved pharmacologically through thrombolytic therapy or mechanically through thrombectomy and stenting procedures. However, despite extensive experimental research, neuroprotective therapies have not yet been successfully translated into routine clinical practice because of limited efficacy and narrow therapeutic windows [8,9].

Ranolazine hydrochloride is a piperazine-derived antianginal agent approved by the U.S. Food and Drug Administration (FDA) for the treatment of chronic stable angina and is recommended as a second-line antianginal therapy in European and North American guidelines [10,11]. Ranolazine primarily inhibits the late sodium current, thereby reducing intracellular sodium and calcium overload, which may contribute to cytoprotective effects under ischemic conditions [12]. Experimental studies have also shown that ranolazine can modulate neuronal sodium channels and reduce neuronal excitability, supporting its potential relevance in CNS-related injury models. Since blood–brain barrier penetration is a critical consideration in CNS research, this pharmacological aspect was also considered when interpreting the rationale and limitations of the present study.

Sirtuins are histone deacetylases involved in regulation of metabolism, DNA transcription, oxidative stress, inflammation, and apoptosis. Among them, Sirtuin 1 (SIRT1) has been shown to suppress NF-κB-mediated inflammatory signaling and inhibit apoptosis through modulation of p53-related pathways [13]. Experimental studies have demonstrated that SIRT1 plays an important role in endogenous neuroprotection during cerebral ischemia through anti-apoptotic and cell-survival mechanisms [14].

The apelinergic system has also emerged as a potential regulator of ischemia-related neuronal injury. Apelin-13 (APL13), one of the most biologically active apelin isoforms, exerts its effects through the apelin receptor (APJ receptor; angiotensin receptor-like 1, APLNR) and is involved in multiple physiological processes, including cardiovascular, neurological, and metabolic regulation [15]. Experimental studies have shown that APL13 reduces neuronal degeneration and apoptosis and exerts neuroprotective effects in models of subarachnoid hemorrhage and cerebral ischemia-reperfusion injury [16,17].

APELA is another endogenous ligand of the APJ receptor with vasodilatory, cardioprotective, anti-inflammatory, and anti-apoptotic properties [18,19]. The APJ receptor is widely expressed in the central nervous system, particularly in neuronal tissue, suggesting a potential role of APELA/APJ signaling in cerebral ischemia and neuroprotection [20].

Beyond its established antianginal effects, ranolazine reduces intracellular sodium accumulation by inhibiting the late sodium current, thereby limiting secondary intracellular calcium overload. Because calcium dysregulation, oxidative stress, mitochondrial dysfunction, inflammation, and apoptosis are key components of ischemia-reperfusion injury, it is conceivable that ranolazine may indirectly influence molecular pathways involved in cellular survival responses. In particular, SIRT1 and the apelinergic system (APL13/APELA–APJ) have both been implicated in anti-apoptotic and neuroprotective signaling during cerebral ischemia. Although a direct interaction between ranolazine and these pathways has not been established, we hypothesized that attenuation of ischemia-related cellular stress responses by ranolazine could be reflected in changes in circulating SIRT1, APL13, and APELA levels.

Therefore, the aim of this study was to evaluate the effects of different doses of ranolazine in a rat middle cerebral artery occlusion/reperfusion (MCAO-I/R) model by investigating histopathological changes and serum levels of SIRT1, APELA, and APL13.

## 2. Materials and Methods

### 2.1. Animals

The study protocol was approved by the Dicle University Animal Experiments Local Ethics Committee (DÜHADEK) (Approval No. DÜHADEK/2024-04; 11 March 2024). In this experimental study, forty-seven male Sprague–Dawley rats weighing 250 ± 20 g were used. During the experimental period, the animals were housed in a quiet, ventilated, and naturally illuminated laboratory environment maintained at 24 ± 2 °C and 50 ± 5% relative humidity under a 12 h light/dark cycle. Rats were fed a standard laboratory diet and housed individually in appropriately sized cages, which were cleaned regularly. Before the experimental procedures, all animals were acclimatized for one week and then randomly allocated into five groups: Sham (*n* = 7), MCAO (*n* = 10), MCAO+RAN10 (ranolazine 10 mg/kg/day, i.p., for 21 days; *n* = 10), MCAO+RAN30 (ranolazine 30 mg/kg/day, i.p., for 21 days; *n* = 10), and MCAO+RAN50 (ranolazine 50 mg/kg/day, i.p., for 21 days; *n* = 10). Ranolazine dihydrochloride (Product No. R0112; TCI, Tokyo Chemical Industry Co., Ltd., Tokyo, Japan) was used in this study.

The selected ranolazine doses (10, 30, and 50 mg/kg/day) were chosen to represent low-, intermediate-, and relatively high-dose exposure in the MCAO-I/R model. This dose range was selected based on previous preclinical studies reporting biological activity of ranolazine in rodents within comparable pharmacological ranges [21,22,23,24,25,26,27]. Inclusion of multiple dose levels enabled assessment of potential dose-dependent effects on ischemia-related histopathological and biochemical outcomes. The highest dose was included to determine whether increased ranolazine exposure would enhance or alter the observed biological responses following cerebral ischemia-reperfusion injury.

Ranolazine was administered intraperitoneally (i.p.) to the treatment groups immediately after MCAO-I/R induction and continued once daily for 20 consecutive days, resulting in a total of 21 administrations. Physiological saline was administered to the Sham and MCAO groups for the same duration.

Rats were anesthetized with ketamine hydrochloride (100 mg/kg) and xylazine hydrochloride (10 mg/kg) prior to both the MCAO procedure and sacrifice. At the end of the experiment (Day 22), approximately 5 mL of whole blood was collected by terminal cardiac puncture under deep anesthesia immediately before sacrifice, corresponding to approximately 21 days after reperfusion. Animals were subsequently euthanized by exsanguination, and brain tissues were immediately harvested and fixed in formaldehyde for histopathological evaluation.

No animals died during the experimental period, and no animals were excluded from the final analyses. All randomized animals completed the study protocol and were included in the histopathological and biochemical evaluations (Figure 1).

### 2.2. Initial Surgical Procedure

For postoperative analgesia, buprenorphine (0.05–0.1 mg/kg, i.p.) was administered at approximately 7 h intervals during the postoperative period as required for pain management. In addition, cefazolin (50 mg/kg, i.m.) was administered as a single preoperative dose for infection prophylaxis. The study design, animal-handling procedures, and reporting were conducted in accordance with the ARRIVE 2.0 guidelines.

### 2.3. Transient Middle Cerebral Artery Occlusion (tMCAO) Model

Transient middle cerebral artery occlusion (tMCAO) was performed according to the method described by Longa et al. [28]. To minimize aspiration-related complications, oral intake was withheld for 4 h preoperatively. Rats were anesthetized with ketamine hydrochloride (100 mg/kg; Ketax^®^; Vem, Istanbul, Turkey) and xylazine hydrochloride (10 mg/kg; Ksilazol^®^; Provet, Istanbul, Turkey) [29]. Body temperature was maintained at 37 ± 0.5 °C throughout the surgical procedure using a heating pad.

Through a midline cervical incision, the right common carotid artery (CCA), external carotid artery (ECA), and internal carotid artery (ICA) were carefully exposed. A small incision was made in the ECA wall, and a silicone-coated nylon monofilament (approximately 0.26–0.28 mm tip diameter) was introduced through the ECA and advanced approximately 18–20 mm beyond the carotid bifurcation into the ICA until mild resistance was felt, indicating occlusion of the origin of the middle cerebral artery (MCA). After 90 min of occlusion, the filament was withdrawn through the ECA to allow for reperfusion.

Successful induction of ischemia and reperfusion was verified using laser Doppler flowmetry. A marked reduction in cerebral blood flow during filament occlusion followed by restoration of flow after filament withdrawal was considered indicative of successful MCAO-I/R induction. During the surgical procedures, physiological parameters including body temperature, heart rate, and oxygen saturation were monitored and maintained within normal physiological ranges.

Rats in the Sham group underwent the same surgical exposure procedure without MCA occlusion.

### 2.4. Serum ELISA Measurements

For APELA, APL13, and SIRT1 analyses, approximately 5 mL of whole blood was collected by terminal cardiac puncture under deep anesthesia on Day 22 (approximately 21 days after reperfusion) immediately before sacrifice and transferred into plain biochemistry tubes. Blood samples were centrifuged at 1500× *g* for 20 min, and the obtained serum samples were stored in Eppendorf tubes at −80 °C until analysis. On the day of analysis, serum samples were thawed at room temperature.

Serum APELA, APL13, and SIRT1 levels were measured using commercially available enzyme-linked immunosorbent assay (ELISA) kits according to the manufacturer’s instructions (Rat APELA ELISA, Catalogue No. 201-11-2091; Rat APL13 ELISA, Catalogue No. 201-11-21771; Rat SIRT1 ELISA, Catalogue No. 201-11-1498; Sunred Biological Technology, Shanghai, China). Measurements were performed using a BioTek ELx50 Microplate Washer and BioTek ELx800 Microplate Reader (BioTek Instruments, Inc., Winooski, VT, USA). Serum APELA and APL13 levels were expressed as pg/mL, and SIRT1 levels as ng/mL.

Serum biomarker analysis was selected because it provides a minimally invasive and clinically relevant approach for evaluating systemic molecular responses following cerebral ischemia. The present study was designed as an exploratory investigation of circulating SIRT1, APELA, and APL13 levels; therefore, tissue-based molecular analyses were not included in the original experimental protocol.

### 2.5. Histopathology

Following completion of the experimental protocol, rats in all groups were sacrificed and brain tissues were harvested for histopathological examination. Whole brains were sectioned into 2 mm thick coronal slices using a microtome blade. Coronal sections of equal thickness were fixed in 10% formaldehyde solution for 48 h and subsequently washed under running tap water to remove residual fixative. Tissue processing was performed using a fully automated tissue processor, and samples were embedded in paraffin blocks.

Sections of 5 µm thickness were cut from the paraffin blocks using a microtome, placed in a water bath, mounted onto positively charged slides, and dried in an incubator. Slides were stained with hematoxylin and eosin using an automated staining system. Histopathological evaluation was performed under light microscopy by a pathologist blinded to group allocation.

Histopathological assessment was performed on coronal brain sections, with the largest ischemic area identified in the third coronal slice. Ischemic changes were observed in the right cerebral hemisphere where ischemia was induced, whereas normal tissue architecture was preserved in the contralateral hemisphere. Nuclear pyknosis, dendritic loss, edema, histiocytic cell infiltration, hemorrhage, apoptotic cells, and calcification were evaluated semi-quantitatively and scored histopathologically.

Neuronal degeneration was evaluated by a blinded pathologist using a semiquantitative ordinal scoring system based on the extent of degenerative neuronal changes observed within the ischemic cortex (0 = absent, 1 = mild, 2 = moderate, 3 = severe degeneration). Apoptotic cells were counted by a blinded pathologist in representative microscopic fields within the ischemic cortex and recorded as the number of apoptotic cells per field. For each animal, counts were performed in multiple representative fields from the area exhibiting the most prominent ischemic changes, and the median value was used for statistical analysis.

### 2.6. Statistical Analysis

Statistical analyses were performed using IBM SPSS Statistics version 21.0 for Windows (IBM Corp., Armonk, NY, USA). Continuous variables were presented as median (minimum–maximum) values, while categorical variables were expressed as frequencies and percentages (%). Data distribution was assessed using the Shapiro–Wilk test. Since the data were not normally distributed, comparisons among the five groups were performed using the Kruskal–Wallis test, followed by Bonferroni-corrected Mann–Whitney U test for pairwise comparisons. All statistical tests were two-tailed, and a *p*-value ≤ 0.05 was considered statistically significant.

## 3. Results

Statistically significant differences were observed among the five groups with respect to apoptotic cell count (*p* = 0.001), neuronal degeneration (*p* < 0.001), serum APELA levels (*p* = 0.018), serum APL13 levels (*p* = 0.011), and serum SIRT1 levels (*p* = 0.007) (Table 1).

### 3.1. Histopathological Findings

In the Sham group, normal cerebral cortex architecture with abundant neurons and neuroglial cells was observed (Figure 2A). In contrast, the MCAO group demonstrated significantly increased apoptotic cell counts (*p* < 0.001) (Figure 3D) and neuronal degeneration scores (*p* < 0.001) (Figure 3E) compared with the Sham group.

Although increased apoptotic cell counts were observed in all ischemia-induced groups relative to the Sham group, pairwise comparisons demonstrated statistically significant differences only in the MCAO and MCAO+RAN50 groups (*p* < 0.001) (Figure 3D). Similarly, neuronal degeneration scores were elevated in all ischemia groups; however, statistically significant differences in pairwise comparisons were observed only in the MCAO and MCAO+RAN50 groups compared with the Sham group (*p* = 0.022) (Figure 3E).

No statistically significant reduction in apoptotic cell counts or neuronal degeneration scores was observed in the ranolazine-treated groups compared with the MCAO group. However, given the relatively small sample size, these findings should be interpreted cautiously and should not be regarded as definitive evidence for the absence of a dose-dependent effect. In addition, a focal area of dystrophic calcification was observed in one rat in the MCAO+RAN50 group (Figure 2I).

### 3.2. ELISA Findings

Blood samples for serum biomarker analyses were collected at the end of the experimental protocol (Day 22), approximately 21 days after reperfusion, corresponding to the chronic phase of ischemia/reperfusion injury.

Serum APELA levels were lower in all ischemia-induced groups compared with the Sham group. However, pairwise comparisons demonstrated a statistically significant decrease only in the MCAO+RAN50 group (*p* = 0.016) (Figure 3A). No statistically significant differences were observed among the remaining groups. In addition, none of the treatment groups showed a statistically significant difference in APELA levels compared with the MCAO group.

For APL13, the highest serum levels were observed in the Sham group, whereas lower levels were detected in the MCAO and ranolazine-treated groups. Pairwise comparisons demonstrated a statistically significant decrease only in the MCAO+RAN50 group compared with the Sham group (*p* = 0.010) (Figure 3C). No significant differences were observed among the remaining groups. Similarly, no statistically significant differences in APL13 levels were detected between the MCAO group and the treatment groups.

Regarding SIRT1, serum levels tended to decrease in the MCAO+RAN30 and MCAO+RAN50 groups and increase in the MCAO+RAN10 group compared with the Sham group; however, these differences did not reach statistical significance. Compared with the MCAO group, a statistically significant decrease in SIRT1 levels was observed only in the MCAO+RAN50 group (*p* = 0.031) (Figure 3B). No statistically significant differences were detected in the remaining treatment groups.

Although several comparisons did not reach statistical significance, numerical differences in biomarker levels were observed among the treatment groups. Given the relatively small sample size, these findings should be interpreted cautiously and warrant further investigation in larger studies designed to evaluate potential dose-dependent effects.

## 4. Discussion

Ischemic stroke is a major neurological disorder that poses a substantial threat to human health worldwide. It results from occlusion of cerebral blood vessels and accounts for approximately 87% of all stroke cases [30]. Current treatment strategies for ischemic stroke primarily focus on reperfusion and neuroprotection. Reperfusion therapies include thrombolytic, antithrombotic, and antiplatelet agents; however, their clinical use is limited by a narrow therapeutic time window and the risk of hemorrhagic complications [31]. Despite extensive experimental research, there is still no safe and effective pharmacological agent routinely available for neuroprotection against ischemia-induced neuronal injury [32].

Because the ischemic penumbra remains potentially salvageable, development of neuroprotective strategies targeting this region continues to be an important focus of experimental stroke research.

Although ranolazine was initially introduced as an immunomodulatory agent, it was later identified as a late sodium channel blocker and approved by the FDA for the treatment of chronic stable angina [10]. Previous studies demonstrating the cytoprotective and potential neuroprotective effects of ranolazine suggest that it may represent a promising candidate for investigation in experimental neuronal injury models [21,22,33]. However, data regarding its effects in isolated cerebral ischemia models remain limited, which prompted the present study.

The MCAO-I/R model is widely used as an experimental model of focal cerebral ischemia because of its relative technical simplicity, its ability to reproduce several pathophysiological features of human ischemic stroke, and its capacity to generate a penumbra region suitable for investigating neuroprotective mechanisms [34].

Following ischemia, neuronal loss occurs within the cerebral parenchyma, and increased apoptosis has been identified as one of the major mechanisms contributing to ischemia-related neuronal injury [5,6,7].

Histopathological examination demonstrated preserved cerebral tissue architecture in the Sham group, with no evidence of hemorrhage or neuronal degeneration. In contrast, the MCAO-I/R groups exhibited ischemia-related pathological changes, including hemorrhage, edema, pyknosis, dendritic loss, neuronal degeneration, and increased apoptosis. Pairwise histopathological comparisons showed a significant increase in apoptotic cell counts in the MCAO group compared with the Sham group, confirming successful induction of ischemic injury (*p* < 0.001).

In the ranolazine-treated ischemia groups, apoptotic changes remained evident compared with the Sham group. A significant increase in apoptotic cell count was observed in the MCAO+RAN50 group compared with the Sham group (*p* < 0.001). Similarly, neuronal degeneration was significantly greater in the MCAO group than in the Sham group (*p* < 0.001). Although neuronal degeneration was observed in all treatment groups, it was most pronounced in the MCAO+RAN50 group, which also showed a significant increase compared with the Sham group (*p* = 0.022).

Gao et al. reported that SIRT1 may contribute to neuroprotection in ischemic injury by reducing neuronal loss through inhibition of apoptosis [14]. Similarly, Razick et al. demonstrated that SIRT1 exerts neuroprotective effects in neurodegenerative diseases by modulating apoptosis-related signaling pathways, thereby reducing neuronal degeneration and slowing disease progression [13]. Although the role of SIRT1 remains complex, most experimental ischemia studies in rats have reported reduced SIRT1 levels following cerebral ischemia. Consistent with these findings, Li et al. demonstrated significantly decreased SIRT1 levels in the ischemia group compared with controls [35].

In our study, serum SIRT1 levels were numerically higher in the MCAO group than in the Sham group; however, this difference was not statistically significant. Therefore, this finding should not be interpreted as a definitive increase in SIRT1 after ischemia. Although several previous studies have reported reduced SIRT1 expression in ischemic brain tissue, differences in sample type, timing of measurement, experimental model, and analytical method may account for the discrepancy observed in serum levels in the present study.

In our study, the highest serum APL13 level was observed in the Sham group. APL13 levels were lower in all ischemia-induced groups, with a decreasing trend observed as ranolazine dosage increased. Overall group comparisons demonstrated a statistically significant difference (*p* = 0.011). Pairwise analysis revealed a significant difference between the Sham and MCAO+RAN50 groups, with the lowest APL13 level detected in the MCAO+RAN50 group (*p* = 0.010).

APL13 has been reported to reduce ischemia-reperfusion injury and provide protection against oxidative damage in various tissues [36]. In a rat MCAO model of focal cerebral ischemia, administration of APL13 during ischemia-reperfusion injury was shown to improve neurological function [37]. Similarly, in experimentally induced subarachnoid hemorrhage, APL13 reduced cortical neuronal degeneration, inhibited apoptosis-related neuronal loss, and exerted neuroprotective effects [16]. In another MCAO ischemia-reperfusion study, exogenous APL13 administration reduced post-ischemic neuronal loss through modulation of multiple signaling pathways associated with apoptosis [17]. In a clinical study, serum APL13 levels were found to be lower in patients with acute ischemic stroke compared with healthy controls, while higher APL13 levels during follow-up were associated with a lower incidence of recurrent stroke [38].

In our study, the lowest APL13 level was measured in the MCAO+RAN50 group, which also exhibited the least favorable histopathological profile among the treatment groups. These findings suggest that high-dose ranolazine was not associated with restoration of APL13-related protective signaling after ischemic injury. However, since SIRT1, APELA, and APL13 were assessed only in serum and not directly in brain tissue, these findings should be interpreted as associative rather than mechanistic. Tissue-level validation using Western blotting, immunohistochemistry, or gene expression analyses would be necessary to clarify whether these circulating biomarker changes reflect local molecular alterations within ischemic cerebral tissue.

APELA is an endogenous peptide that shares structural and functional similarities with apelin and has been implicated in angiogenesis, fluid homeostasis, cell regeneration, and growth [39].

In our study, serum APELA levels were measured during the chronic phase, and significant differences were observed among the groups (*p* = 0.018). Pairwise comparisons demonstrated a significant difference between the Sham and MCAO+RAN50 groups, with the lowest APELA level detected in the highest-dose ranolazine group (*p* = 0.016). Although only one pairwise comparison reached statistical significance, APELA levels generally tended to be lower in the ischemia groups than in the Sham group. In addition, APELA levels in the ranolazine-treated groups showed a dose-dependent decreasing trend compared with the MCAO group.

Xu et al. reported that in a rat MCAO ischemia-reperfusion model, serum APELA levels increased during the first 24 h after ischemia and were higher than those in the control group. However, serial measurements demonstrated a progressive decline in APELA levels during the later stages of ischemia-reperfusion injury. These findings suggest that serum APELA levels may exhibit different patterns during the acute and chronic phases of cerebral ischemia. In the same study, exogenous APELA administration exerted neuroprotective effects through inhibition of ferroptosis in ischemic groups [40].

In our study, APELA levels were generally low, which may partly be related to measurements being performed during the chronic phase of ischemia. In addition, APELA levels in the treatment groups were lower than those in the Sham group, with the lowest level observed in the MCAO+RAN50 group. Although these findings suggest an unfavorable biochemical pattern in the highest-dose ranolazine group, the absence of serial measurements and tissue-based validation limits mechanistic interpretation. Therefore, these results should not be considered definitive evidence of ranolazine-induced neurodegeneration.

Siddiqui et al. reported neuroprotective effects of ranolazine in an MCAO model, where oral ranolazine administered in combination with clopidogrel before reperfusion and continued for 14 days reduced infarct volume, improved cerebral blood flow and neurological function, and attenuated apoptosis [23]. However, several methodological differences should be considered when comparing their findings with ours. In the study by Siddiqui et al., ranolazine was administered orally in combination with clopidogrel, whereas in our study it was administered intraperitoneally, as monotherapy, and for a longer duration (21 days). Differences in treatment timing and experimental design may also have influenced the outcomes. These methodological differences may partly explain the discrepancy between findings and suggest that the effects of ranolazine in cerebral ischemia may vary depending on administration route, treatment duration, timing, and concomitant therapies.

Ebrahimpour et al. highlighted the potential of ranolazine to reduce AGE-related injury and oxidative stress in diabetic cardiomyopathy independent of glucose-lowering effects [24]. Chunchai et al. demonstrated that ranolazine alleviated doxorubicin-induced neurotoxicity in an experimental diabetes model, suggesting a potential neuroprotective effect in addition to pancreatic protection [22]. Similarly, Cappetta et al. reported that ranolazine improved diastolic dysfunction and preserved cardiac function in a doxorubicin-induced cardiotoxicity model through inhibition of the late Na^+^ current [33]. Previous studies have suggested that the primary mechanism of ranolazine involves blockade of late sodium channels, resulting in reduced intracellular calcium accumulation and modulation of downstream enzymatic processes [25,26,27].

Although previous studies have demonstrated cytoprotective effects of ranolazine through oxidative stress-related and tissue-specific mechanisms, our findings did not demonstrate a clear neuroprotective effect in the MCAO-I/R model. None of the ranolazine-treated groups showed convincing histopathological improvement compared with the untreated MCAO group, and the highest-dose group exhibited the least favorable biomarker profile, including lower SIRT1, APL13, and APELA levels. The present study was not designed to demonstrate a direct mechanistic interaction between ranolazine and SIRT1 or apelin/APJ signaling pathways. Rather, these biomarkers were selected because of their previously reported involvement in ischemia-related apoptosis, oxidative stress, and neuroprotection. Therefore, the observed biomarker changes should be interpreted as exploratory associations rather than definitive evidence of direct pathway modulation by ranolazine. However, the present data are more consistent with absence of neuroprotection and unfavorable findings at the highest dose rather than definitive evidence of a direct neurodegenerative effect.

Because serum biomarker measurements were performed only at a single late time point during the chronic phase, the present findings may not fully reflect acute ischemia-related molecular responses. In addition, the absence of parallel brain tissue-based analyses limits direct correlation between circulating biomarker levels and local neuronal injury. Therefore, absence of early-phase measurements limits interpretation of the present data with respect to acute neuroprotective mechanisms.

## 5. Study Limitations

Several limitations of this study should be acknowledged. Infarct volume assessment using TTC (2,3,5-triphenyltetrazolium chloride) staining or imaging-based approaches was not performed in the present study. TTC staining is most reliable during the acute and subacute phases of cerebral ischemia, whereas the present experimental design focused on chronic-phase evaluation following 21 days of treatment. Because delayed tissue remodeling, cavitation, and gliosis may reduce the reliability of TTC-based infarct delineation in chronic settings, this method was not included. Nevertheless, the absence of infarct volume quantification represents an important limitation and restricts comprehensive evaluation of ischemic injury severity and treatment efficacy. Future studies should incorporate infarct volume analysis using appropriate acute-phase TTC protocols or advanced imaging and histomorphometric techniques [41].

Immunohistochemical staining, Western blot analysis, and gene expression studies for SIRT1, APELA, and APL13 in ischemic brain tissue were not included in the present study design. These tissue-level molecular analyses could have provided important mechanistic insights regarding local protein expression and signaling pathway alterations after ischemia-reperfusion injury. Furthermore, serum biomarker levels may not fully reflect molecular events occurring within cerebral tissue. Therefore, the mechanistic interpretation of the present findings remains limited, and future studies should integrate serum, tissue-based, and molecular analyses for a more comprehensive evaluation.

The absence of isoform-specific analyses, particularly for SIRT1, may represent an additional limitation, as stress conditions are known to alter isoform distribution, which cannot be fully captured by ELISA-based measurements [42].

A further limitation is that an a priori statistical power analysis was not performed. The sample size was determined based on ethical considerations, feasibility, and group sizes commonly used in experimental MCAO studies. Although statistically significant differences were detected for several histopathological and biochemical parameters, the relatively small sample size may have limited the ability to detect more subtle treatment-related effects, particularly in pairwise comparisons among ranolazine-treated groups. Therefore, the present findings should be interpreted as preliminary, and future studies with a priori power calculations and larger sample sizes are required to confirm these results.

Another important limitation is the absence of neurological and behavioral functional assessments. Standardized neurological deficit scoring systems and motor function tests are widely used in MCAO studies to evaluate functional recovery and treatment efficacy. The lack of these assessments limits the ability to correlate biochemical and histopathological findings with neurological outcomes.

## 6. Conclusions

The MCAO+RAN50 group demonstrated lower serum SIRT1, APELA, and APL13 levels compared with several other groups. However, given the limited sample size, absence of infarct volume analysis, lack of neurological functional assessment, and absence of tissue-level molecular validation, these findings should be interpreted cautiously and should not be considered definitive evidence of a harmful effect of high-dose ranolazine.

Future studies incorporating functional outcomes, infarct volume assessment, tissue-based molecular analyses, and translational experimental designs are needed to clarify the potential clinical relevance of ranolazine in ischemic stroke and to determine whether specific treatment regimens may provide neuroprotective benefit.

## Figures and Tables

**Figure 1:**
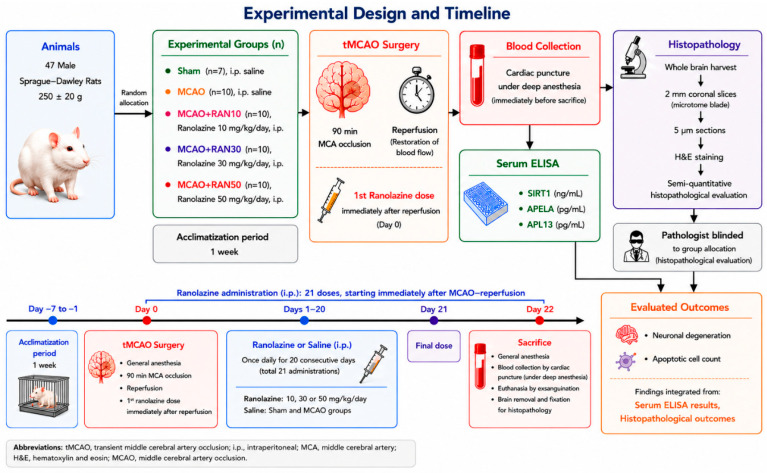
Experimental design and timeline.

**Figure 2:**
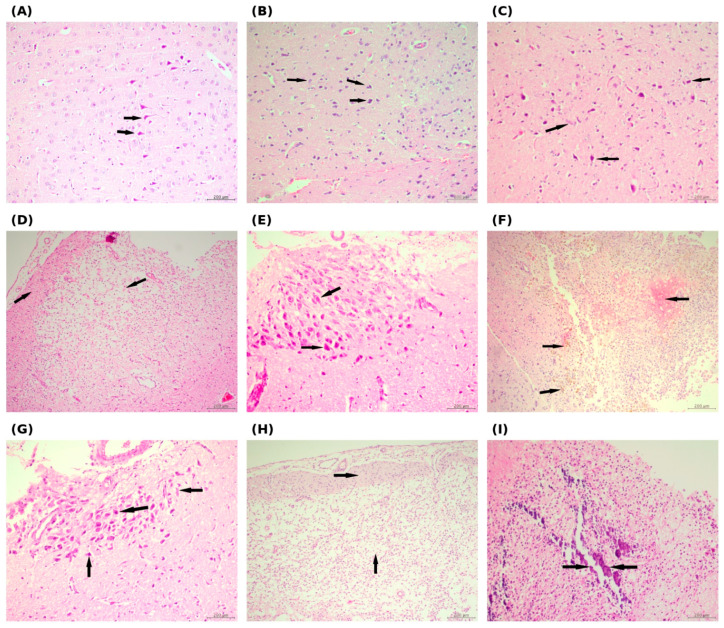
Representative histopathological findings in cerebral cortex sections following middle cerebral artery occlusion/reperfusion (MCAO-I/R) and ranolazine treatment (hematoxylin and eosin staining). (**A**,**B**) Sham group demonstrating preserved cerebral cortical architecture, normal neuronal morphology, and absence of ischemia-related pathological changes. (**C**) MCAO group showing edema and disruption of normal tissue architecture. (**D**) MCAO group demonstrating pyknotic/apoptotic neurons and neuronal degeneration. (**E**) MCAO group showing hemorrhagic and inflammatory changes within the ischemic region. (**F**) MCAO+RAN10 group demonstrating persistent ischemia-related neuronal degeneration. (**G**) MCAO+RAN30 group showing edema and degenerative neuronal changes. (**H**) MCAO+RAN50 group demonstrating apoptotic morphology and neuronal injury. (**I**) Focal dystrophic calcification observed in one rat from the MCAO+RAN50 group. Arrows indicate representative pathological findings. Scale bar = 200 μm.

**Figure 3:**
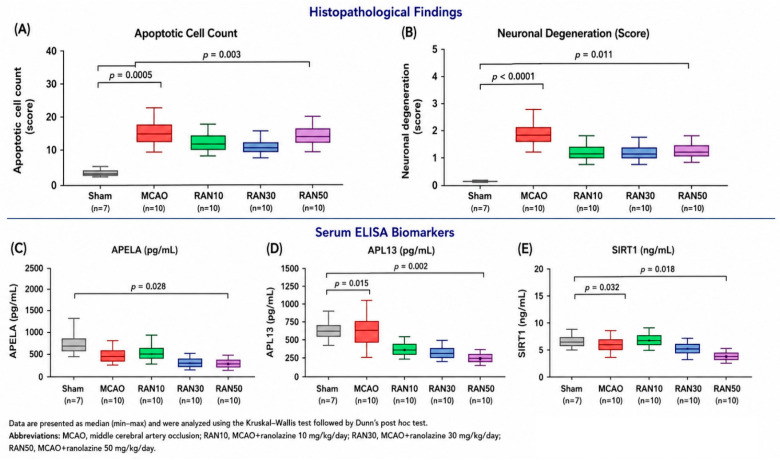
Quantitative histopathological and serum biomarker analyses among experimental groups. (**A**) Apoptotic cell count score. (**B**) Neuronal degeneration score. (**C**) Serum APELA levels. (**D**) Serum APL13 levels. (**E**) Serum SIRT1 levels. Data are presented as median and distribution range using box-and-whisker plots. Statistical analyses were performed using the Kruskal–Wallis test followed by Bonferroni-corrected Mann–Whitney U test for pairwise comparisons. Exact statistically significant pairwise comparisons and *p*-values are indicated within the figure panels. Abbreviations: MCAO, middle cerebral artery occlusion; RAN10, MCAO+ranolazine 10 mg/kg/day; RAN30, MCAO+ranolazine 30 mg/kg/day; RAN50, MCAO+ranolazine 50 mg/kg/day.

**Table 1:** Comparison of serum biomarker levels and histopathological findings among study groups.

Parameter	Sham (*n* = 7)	MCAO (*n* = 10)	MCAO+RAN10 (*n* = 10)	MCAO+RAN30 (*n* = 10)	MCAO+RAN50 (*n* = 10)	*p* Value
APELA (pg/mL)	433.93 (314.45–1515.75)	378.89 (291.88–611.72)	360.07 (265.54–923.10)	304.57 (242.96–404.76)	283.41 (201.57–521.41) *	0.018
APL13 (pg/mL)	302.11 (201.16–484.19)	260.99 (149.54–1224.95)	210.23 (175.06–276.59)	196.33 (177.90–241.99)	168.25 (148.41–212.50) *	0.011
SIRT1 (ng/mL)	5.27 (4.57–12.67)	6.01 (3.62–11.61)	5.83 (4.83–10.96) †	4.79 (4.31–7.28)	4.20 (3.25–4.76) #†	0.007
Apoptotic cell count	4.00 (3–5)	18.50 (11–22) *	11.50 (10–24)	12.50 (10–16)	15.00 (9–22) *	0.001
Degree of neuronal degeneration	0 (0–0)	2 (1–3) *	1 (1–2)	1 (1–2)	1 (1–2) *	<0.001

Data are presented as median (min–max). Comparisons among groups were performed using the Kruskal–Wallis test followed by Bonferroni-corrected Mann–Whitney U test for pairwise comparisons. Symbols indicate statistically significant pairwise differences within the same row: * *p* < 0.05 versus Sham group; # *p* < 0.05 versus MCAO group; † *p* < 0.05 versus MCAO+RAN10 group.

## Data Availability

The original contributions presented in this study are included in the article. Further inquiries can be directed to the corresponding author.

## References

[1] Feigin V.L., Brainin M., Norrving B., Martins S.O., Pandian J., Lindsay P., Grupper M.F., Rautalin I. (2025). World Stroke Organization: Global Stroke Fact Sheet 2025. Int. J. Stroke.

[2] Margetis K., Sánchez-Manso J.C. (2025). Neuroanatomy, Middle Cerebral Artery. StatPearls.

[3] Deb P., Sharma S., Hassan K.M. (2010). Pathophysiologic mechanisms of acute ischemic stroke: An overview with emphasis on therapeutic significance beyond thrombolysis. Pathophysiology.

[4] Markus H.S. (2004). Cerebral perfusion and stroke. J. Neurol. Neurosurg. Psychiatry.

[5] Babić A., Bonifačić D., Komen V., Kovačić S., Mamić M., Vuletić V. (2025). Blood Biomarkers in Ischemic Stroke Diagnostics and Treatment-Future Perspectives. Medicina.

[6] Seetge J., Frenger J., Katan M., Grosse G.M. (2026). Recent advances in stroke biomarkers—Implications for prognosis and treatment. Curr. Opin. Neurol..

[7] Lee J., Giannaris P.S., Yilmaz C.E., Yilmaz G. (2025). Emerging biomarkers in ischemic stroke. Vessel Plus.

[8] Di Lorenzo R., Saqqur M., Buletko A.B., Handshoe L.S., Mulpur B., Hardman J., Donohue M., Wisco D., Uchino K., Hussain M.S. (2021). IV tPA given in the golden hour for emergent large vessel occlusion stroke improves recanalization rates and clinical outcomes. J. Neurol. Sci..

[9] Devos D., Sevin M., De Gaalon S., Lintia-Gaultier A., Guillon B. (2013). Management of ischemic stroke in the hyperacute phase. Pannminerva Med..

[10] Montalescot G., Sechtem U., Achenbach S., Andreotti F., Arden C., Budaj A., Bugiardini R., Crea F., Cuisset T., Task Force Members (2013). 2013 ESC guidelines on the management of stable coronary artery disease: The Task Force on the management of stable coronary artery disease of the European Society of Cardiology. Eur. Heart J..

[11] Fihn S.D., Gardin J.M., Abrams J., Berra K., Blankenship J.C., Dallas A.P., Douglas P.S., Foody J.M., Gerber T.C., Hinderliter A.L. (2012). 2012 ACCF/AHA/ACP/AATS/PCNA/SCAI/STS guideline for the diagnosis and management of patients with stable ischemic heart disease: A report of the American College of Cardiology Foundation/American Heart Association task force on practice guidelines, and the American College of Physicians, American Association for Thoracic Surgery, Preventive Cardiovascular Nurses Association, Society for Cardiovascular Angiography and Interventions, and Society of Thoracic Surgeons. Circulation.

[12] Zhang J., Yan B., Wang J. (2025). Development trend analysis of ranolazine, based on bibliometrics. Medicine.

[13] Razick D.I., Akhtar M., Wen J., Alam M., Dean N., Karabala M., Ansari U., Ansari Z., Tabaie E., Siddiqui S. (2023). The Role of Sirtuin 1 (SIRT1) in Neurodegeneration. Cureus.

[14] Gao H., Yang L., Shao Y. (2022). SIRT1/NF-κB pathway on neuronal apoptosis in rats with ischemic stroke. Cell. Mol. Biol..

[15] Hu G., Wang Z., Zhang R., Sun W., Chen X. (2021). The Role of Apelin/Apelin Receptor in Energy Metabolism and Water Homeostasis: A Comprehensive Narrative Review. Front. Physiol..

[16] Shen X., Yuan G., Li B., Cao C., Cao D., Wu J., Li X., Li H., Shen H., Wang Z. (2022). Apelin-13 attenuates early brain injury through inhibiting inflammation and apoptosis in rats after experimental subarachnoid hemorrhage. Mol. Biol. Rep..

[17] Shao Z.Q., Dou S.S., Zhu J.G., Wang H.Q., Wang C.M., Cheng B.H., Bai B. (2021). Apelin-13 inhibits apoptosis and excessive autophagy in cerebral ischemia/reperfusion injury. Neural Regen. Res..

[18] Zhang Y., Wang Y., Lou Y., Luo M., Lu Y., Li Z., Wang Y., Miao L. (2018). Elabela, a newly discovered APJ ligand: Similarities and differences with Apelin. Peptides.

[19] Xu C. (2021). The Elabela in hypertension, cardiovascular disease, renal disease, and preeclampsia: An update. J. Hypertens..

[20] Luo H., Han L., Xu J. (2020). Apelin/APJ system: A novel promising target for neurodegenerative diseases. J. Cell. Physiol..

[21] Yue X., Wu S., Yin Y., Zhao X., Li E., Liu G., Zan X., Yu Q., He P., Zhang C. (2025). The novel effects of the cardiovascular drug ranolazine on the alleviation of age-related cognitive decline and the underlying mechanisms. Eur. J. Pharmacol..

[22] Chunchai T., Arinno A., Ongnok B., Pantiya P., Khuanjing T., Prathumsap N., Maneechote C., Chattipakorn N., Chattipakorn S.C. (2022). Ranolazine alleviated cardiac/brain dysfunction in doxorubicin-treated rats. Exp. Mol. Pathol..

[23] Siddiqui M.A., Pathak A., Ramakrishna K., Krishnamurthy S. (2025). Ranolazine neuroprotection against middle cerebral artery occlusion/reperfusion ischemic injury via modulation of brain-derived neurotrophic factor and brain mitochondrial tu translation elongation factor (TUFM). Metab. Brain Dis..

[24] Ebrahimpour F., Bathaie S.Z., Yaghooti H. (2026). Ranolazine attenuates diabetic cardiomyopathy in rats by suppressing AGE-RAGE axis and oxidative stress. BMC Res. Notes.

[25] Singh D., Oladimeji-Salami J.A., Akindele A.J. (2025). Unraveling the pharmacological and therapeutic potential of Ranolazine beyond antianginal drug use: A new insight. Exp. Biol. Med..

[26] Kayan F., Savas H.B. (2025). Effects of ranolazine on angiogenesis and oxidant-antioxidant balance: An in vivo experimental model study. Sci. Rep..

[27] Tentolouris K.N., Anastasiou I.A., Mourouzis I., Pantos C., Tentolouris N. (2025). Ranolazine: An Established Anti-anginal Drug with Emerging Antidiabetic Potential Supported by Preclinical and Clinical Evidence. Cardiovasc. Hematol. Disord. Drug Targets.

[28] Longa E.Z., Weinstein P.R., Carlson S., Cummins R. (1989). Reversible middle cerebral artery occlusion without craniectomy in rats. Stroke.

[29] Chavda V., Patel S. (2022). Voglibose and saxagliptin ameliorate the post-surgical stress and cognitive dysfunction in chronic anaesthesia exposed diabetic MCAo induced ischemic rats. IBRO Neurosci. Rep..

[30] Benjamin E.J., Muntner P., Alonso A., Bittencourt M.S., Callaway C.W., Carson A.P., Chamberlain A.M., Chang A.R., Cheng S., Das S.R. (2019). Heart Disease and Stroke Statistics-2019 Update: A Report From the American Heart Association. Circulation.

[31] Powers W.J., Rabinstein A.A., Ackerson T., Adeoye O.M., Bambakidis N.C., Becker K., Biller J., Brown M., Demaerschalk B.M., Hoh B. (2019). Correction to: Guidelines for the Early Management of Patients with Acute Ischemic Stroke: 2019 Update to the 2018 Guidelines for the Early Management of Acute Ischemic Stroke: A Guideline for Healthcare Professionals from the American Heart Association/American Stroke Association. Stroke.

[32] Paul S., Candelario-Jalil E. (2021). Emerging neuroprotective strategies for the treatment of ischemic stroke: An overview of clinical and preclinical studies. Exp. Neurol..

[33] Tantray J., Menezes-Rodrigues F.S., Podanosque J.M., Tallo F.S., Caricati-Neto A., Sharma A.K., Patel A., Sharma R.K., Singh S., Beibituly B.A. (2026). Cardioprotective effects of the ranolazine in myocardial infarction mediated by stimulation of the endogenous mediators involved in ischemic preconditioning. Acta Cir. Bras..

[34] Heras-Romero Y., Morales-Guadarrama A., Olayo-Gonzalez R., Roldan-Valadez E. (2026). Practical Guide and Narrative Review of the Rat MCAO Model in Preclinical Stroke: Surgical Standards and MRI/DTI/MRS Biomarkers. Curr. Neuropharmacol..

[35] Li Z., Xing J. (2025). Role of sirtuins in cerebral ischemia-reperfusion injury: Mechanisms and therapeutic potential. Int. J. Biol. Macromol..

[36] Guo P., Siddique R., Qian J., Qi L., Yong V.W., Xue M. (2025). Apelin-13 Attenuates Blood-Brain Barrier Dysfunction Following Intracerebral Hemorrhage via Targeting the Keap1/Nrf2 Signaling. CNS Neurosci. Ther..

[37] Oruc K.Y., Oruc A., Arslan R., Diriarin F.P., Mengi M., Tanriverdi G., Yanar K., Ozeren Eser M., Agturk G., Sonkurt A.I. (2026). Apelin-13 confers Neuropeptide Y-mediated neuroprotection and preserves learning and allocentric memory in D-glutamic acid-induced excitotoxicity in rats. Mol. Neurobiol..

[38] Jia H., Zhao Q., Yuan J., Wang X., Chen H., Dong J., Zhu C., Maslov L.N., Naryzhnaya N.V., Guan Y. (2026). Apelin-13 ameliorates myocardial ischemia/reperfusion injury by modulating macrophage polarization. Arch. Biochem. Biophys..

[39] Li W., Meng C., Yang S., Xie Y., Huang Z., Wang T. (2026). ELABELA: A Potential Therapeutic Target for Ischemia-Reperfusion Injury. Biomolecules.

[40] Xu P., Kong L., Tao C., Zhu Y., Cheng J., Li W., Shen N., Li R., Zhang C., Wang L. (2023). Elabela-APJ axis attenuates cerebral ischemia/reperfusion injury by inhibiting neuronal ferroptosis. Free. Radic. Biol. Med..

[41] Poinsatte K., Selvaraj U.M., Ortega S.B., Plautz E.J., Kong X., Gidday J.M., Stowe A.M. (2015). Quantification of Neurovascular Protection Following Repetitive Hypoxic Preconditioning and Transient Middle Cerebral Artery Occlusion in Mice. J. Vis. Exp..

[42] Hwang J.W., Yao H., Caito S., Sundar I.K., Rahman I. (2013). Redox regulation of SIRT1 in inflammation and cellular senescence. Free. Radic. Biol. Med..

